# Whole-Body Dynamics-Based Aerial Fall Trajectory Optimization and Landing Control for Humanoid Robot

**DOI:** 10.3390/biomimetics8060460

**Published:** 2023-10-01

**Authors:** Weilong Zuo, Junyao Gao, Jingwei Cao, Xilong Xin, Mingyue Jin, Xuechao Chen

**Affiliations:** 1 School of Mechatronical Engineering, Beijing Institute of Technology, Beijing 100081, China; 2 Beijing Advanced Innovation Center for Intelligent Robotics and Systems, Beijing Institute of Technology, Beijing 100081, China

**Keywords:** fall, trajectory optimization, control, humanoid robot

## Abstract

When humanoid robots work in human environments, falls are inevitable due to the complexity of such environments. Current research on humanoid robot falls has mainly focused on falls on the ground, with little research on humanoid robots falling from the air. In this paper, we employ an extended state variable formulation that directly maps from the high-level motion strategy space to the full-body joint space to optimize the falling trajectory in order to protect the robot when falling from the air. In order to mitigate the impact force generated by the robot’s fall, during the aerial phase, we employ simple proportion differentiation (PD) control. In the landing phase, we optimize the optimal contact force at the contact point using the centroidal dynamics model. Based on the contact force, the changes to the end-effector positions are solved using a dual spring–damper model. In the simulation experiments, we conduct three comparative experiments, and the simulation results demonstrate that the robot can safely fall 1.5 m from the ground at a pitch angle of 45°. Finally, we experimentally validate the methods on an actual robot by performing a side-fall experiment. The experimental results show that the proposed trajectory optimization and motion control methods can provide excellent shock absorption for the impact generated when a robot falls.

## 1. Introduction

Humanoid robots are a tangible manifestation of human technological advancement, making them of great interest to researchers. The world’s most advanced humanoid, Atlas, demonstrates flexible motion and manipulation abilities, pointing to the current direction of humanoid robotics development [1]. Business magnate Musk has greatly supported humanoid robotic technology by funding relevant research. As seen in the latest video [2], Optimus can now assist humans in performing tasks in human environments.

At present, humanoid robot research primarily centers on walking, running, jumping, and aiding people in performing duties [3,4,5,6]. Due to the complexity of the human environment, robots will inevitably experience falling behavior when completing these tasks. Therefore, designing a structure or control method that can effectively protect robots is very important. According to the law of impulse, when the robot has a certain mass, reducing the impact force of the robot on the ground mainly involves increasing the touchdown time and reducing the landing speed. For the aspect of increasing the ground impact time, Kajita et al. introduced a method using airbags [7]; however, the airbags employed can only be used once and must reinflate before they can be used again, making the method complex and expensive. Similarly, Sung-Hee Lee introduced a way of placing a backpack on the robot’s back [8] to change the falling direction after the robot receives an impact, which is suitable for environments wherein surrounding objects do not interfere. In [9], Kakiuchi designed a robot with hard points mounted over its entire body, which looks like a man wearing a set of shackles and significantly limits the robot’s workspace. Inspired by the self-protection of turtles, Nguyen et al. designed a type of shell protector. However, in their latest research, since the robot only had a lower body, the actual fall performance of the whole body has not yet been observed [10]. In the aspect of decreasing the ground impact velocity, researchers use methods for optimizing trajectories and motion control methods. For example, Fujiawara optimized the falling trajectories of robots in different orientations through imitating Ukemi motion by using energy shaping and distribution methods [11]. Luca established a variable-length inverted pendulum model to optimize forward falling trajectories for a robot [12]. In addition, some researchers optimize the the distribution of contact points and the contact sequence, as described in [13,14]. Regarding robot motion control, being able to effectively cushion impact forces is a very important strategy. Mujica proposed a variable admittance control that successfully achieved human–robot collaborative tasks [15]. For the task of adjusting control parameters, ref. [16] proposed a new Lyapunov-based offline self-tuning control method. Similarly to [15,17] presented a compliant adaptive control method that can enable stable and safe physical human–robot interaction during work. In recent research, MIT proposed using model predictive control to mitigate the impact forces of acrobatic robot maneuvers [18]. HaoxiangQi achieved stable control after a robot’s high jump landing by optimizing contact forces and using a virtual model [19].

Based on the above references, we can see that current research on robot falls mainly considers robots falling on flat ground. However, research on robots falling from high altitudes is still lacking. Because human exploration desires are endless, people also hope that robots can replace humans to accomplish some high-difficulty actions such as aerial acrobatics and parachuting. But when performing these actions, robots face the risk of falling. Therefore, more research needs to focus on solutions to help robots mitigate the impact of falling from high altitudes in order to achieve complex aerial tasks that are difficult for humans.

**Figure 1:**
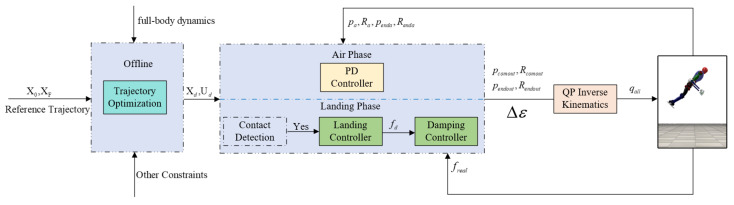
Planning and control framework for a humanoid robot falling from the air. The superscripts “d” and “a” mean the desired value and the actual (measured) value, respectively; $X_{0}$ and $X_{F}$ represent the initial state and the final state, respectively; $X_{d}$ and $U_{d}$ represent the expected state and the expected input value, respectively.

Research on falling mitigation for robots mainly focuses on quadruped robots, notably the Mini Cheetah from MIT [20,21]. Research employs offline or online optimization to combine machine learning or neural networks when falling from heights. The results show that by using the method in [20], a robot can fall from a height of 10 feet (about 3 m); while using the method in [21], a robot can fall from around a 2 m height. Another avenue of research is from a bio-inspired perspective, where a tail is added to the quadruped robot to cushion the impact of falling, which is discussed in papers [22,23]. Different from the methods and experimental objectives mentioned above, Francesc proposed an optimization-based reactive landing controller for handling horizontal impacts during falls [24] and showed that a quadruped robot can recover from falls at horizontal speeds up to 3 m/s. In this paper, we adopted a direct mapping relationship from the high-level motion task strategy space to the robot’s whole joint space by setting the robot’s different state variables and adding the corresponding contact constraint equations. In this method, the high-level action task strategy is utilized as the objective function of the robot, allowing the optimizer to satisfy our desired actions. For instance, when a robot experiences a fall from the air, its high-level motion task strategy space is to minimize contact force, follow a prior trajectory function, and distribute the force evenly across each contact point. Using the optimizer solution method, we optimized the trajectory of a robot falling from the air given the initial and target states. When the robot was in the aerial stage, we used a proportion derivative (PD) method to control the posture of the centroid and endpoints. When the robot was in the landing stage, the target reference force was optimized using centroid dynamics to reduce the impact force by damping control. Figure 1 illustrates the overall framework of our method.

The contribution of this work is three-fold:

- An optimized trajectory for the robot falling from the air is generated, enabling the humanoid robot to land smoothly on the ground.
- Direct mapping is adopted from the high-level task strategy space to the joint space, bypassing the need to go through the centroid space as in previous methods.
- Simulation results show that the trajectories optimized after adding contact point information are more human-like compared to those without contact information.

## 2. Trajectory Optimization

**Figure 2:**
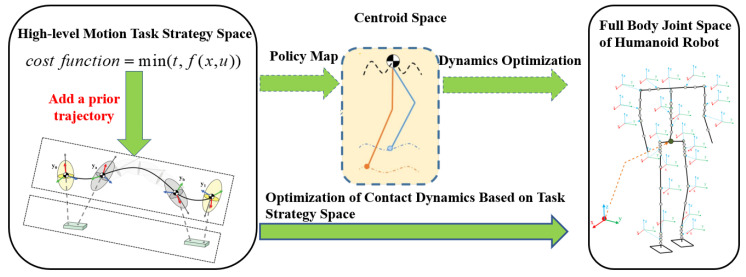
The differences between step-by-step centroid space optimization and unified strategy space planning methods.

Traditional motion planning methods map from the high-level motion task strategy space to the centroid space and then to the full-body joint space of humanoid robots [25]. The advantage of this method is that the centroid motion model is simple and can easily generate whole-body joint motions. However, due to the oversimplified movements in the centroid space, which cannot consider all possible contacts, and its inability to utilize known information about the robot’s spatial posture, we employ an optimization method that maps directly from the high-level motion task strategy space to the full-body joint space of the robot in this paper, as shown in Figure 2.

### 2.1. Methods

According to the above-mentioned method, we adopt an approach that works from the high-level task space directly to the joint space, putting the robot’s joint angles into the optimization variables. The state variables of existing trajectory optimization contact models mainly focus on the position, direction, velocity, and angular velocity of the center of mass; and since a humanoid robot produces contact forces with the ground during the landing phase, these contact forces also have an impact on the robot’s overall state, so this paper considers the robot’s contact forces and the velocity and energy of the contact point and establishes the following form of state variables:

(1) $$x={\left[p_{com};\theta_{com};q_{all}\right]}^{T}$$

(2) $$X={\left[x;\dot{x};p_{c};\dot{p_{c}};W_{c1}\ldots W_{cn}\right]}^{T}$$

where $p_{com}$ represents the position of the centroid, $\theta_{com}$ represents the direction of the centroid, $q_{all}$ represents all joint angles, $p_{c}$ represents the position of the contact point, ${\dot{p}}_{c}$ represents the velocity of the contact point, and $W_{ci}$ represents the work done by the contact force corresponding to the *i*-th contact point.

Here, we represent the control input of the robot as follows

(3) $$U={\left[\tau_{i};f_{1}\ldots f_{n}\right]}^{T}$$

where τ represents the driving torque of the corresponding joint of the robot, and $f_{n}$ represents the contact force of the n-th contact point. The advantages of using this form of state variables and control inputs are: First, we added the state update equations for the contact point position dimensions, allowing us to leverage partial prior information about contact points from the reference trajectory. Second, the additional terms for contact force work nicely to avoid energy conservation issues while facilitating reference trajectories based on energy changes. To further validate that the state variables proposed in this paper can provide good guidance for trajectory optimization of the robot’s falling state, we conducted a comparison in the simulation section to demonstrate its effectiveness.

When a humanoid robot falls from the air, it has no contact with the environment, but when it lands on the ground, parts of its body come into contact with the surroundings. These contact forces affect the robot’s base; however, the full-body dynamic model provides equations describing the impact of external forces on the robot’s motion. According to reference [26], we can obtain the robot dynamic equation involving all external contact forces as

(4) $$D\left(x\right)\ddot{x}+C\left(x,\dot{x}\right)\dot{x}+G\left(x\right)=\tau +\sum_{i=1}^{n}J_{ci}^{T}f_{i}$$

where $D\left(x\right)\in R^{\left(N+6)\times (N+6\right)}$ is the mass inertia matrix, $C\left(x,\dot{x}\right)\in R^{\left(N+6)\times (N+6\right)}$ is Coriolis force, $G\left(x\right)\in R^{\left(N+6)\times (N+6\right)}$ is gravity acting on the robot, $\tau \in R^{(N+6)\times 1}$ are the joint torques of the robot, $x\in R^{(N+6)\times 1}$ are the state variables of the robot, $J_{ci}\in R^{\left(N+6)\times (6n)\right)}$ is the Jacobean matrix of the i-th contact point, and $f_{i}\in R^{(6n)\times 1}$ is the contact force of the robot at the i-th contact point, where *N* denotes the number of degrees of freedom. When a robot’s body comes into contact with the ground, an acceleration is produced at the point of contact. According to the contact dynamics equation mentioned above, the acceleration at the contact point can be related to the contact force via an expression as follows

(5) $${\ddot{p}}_{c}={\dot{J}}_{c}\dot{x}+J_{c}D^{-1}\left(u-C\left(x,\dot{x}\right)\dot{x}-G\left(x\right)-\sum_{i=1}^{n}J_{i}^{T}f_{i}\right)$$

### 2.2. Cost Function

In this paper, we aim to achieve the reference target with minimal driving force, and there should be no uneven force distribution at each contact point. Therefore, the objective function can be defined as follows:

(6) $$J=\int_{t_{0}}^{t_{f}}w_{1}{\left(U-U_{ref}\right)}^{2}+\int_{t_{0}}^{t_{f}}w_{2}{\left(X-X_{ref}\right)}^{2}+\int_{t_{0}}^{t_{f}}w_{3}{\left(F_{c}-F_{ref}\right)}^{2}$$

where *U* represents the robot’s control input; $U_{ref}$ represents the robot’s reference input; *X* and $X_{ref}$ represent the state variables and reference variables mentioned earlier, respectively; $F_{ref}$ represents the reference contact force; and $w_{1}$, $w_{2}$, and $w_{3}$ represent the corresponding weight matrices. The purpose of setting the third term is that we do not expect the robot to have an uneven distribution of forces after falling down the ground. In optimization, we expect the robot’s knees and arms to land, resulting in a $F_{ref}$ value of mg/4. In addition, we expect the robot’s center of mass to be 0.8 m above the ground with no slippage at the contact points and with the robot’s roll, pitch, and yaw angles to be 0.

### 2.3. Constraints

**Figure 3:**
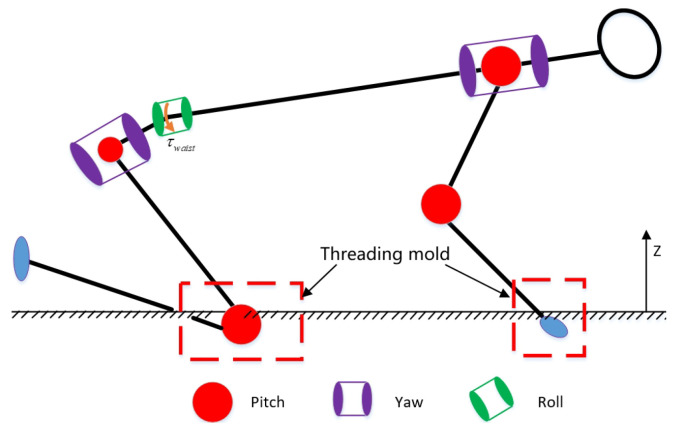
The robot exhibits ground penetration. In the simulation, the robot is in a non-dynamic environment with its arms and knees already penetrating the ground.

The following constraints are crucial for optimizing the trajectory:

(1). At the start of optimizing the robot, the minimum and maximum values need to be specified for the initial state variable $X_{0}$; also, the minimum and maximum desired values for the final state variable $X_{F}$ need to be specified. Additionally, the minimum time $t_{min}$ and maximum time $t_{max}$ for the desired robot motion, the minimum $q_{min}$ and maximum $q_{max}$ values for all joints, and the lower limit $U_{min}$ and upper limit $U_{max}$ for the control inputs need to be specified. To accelerate convergence, an initial guess value is also created, including the motion time $t_{guess}$, state $X_{guess}$, and control input $U_{guess}$ for the robot.

(2). For the dynamics, we want the entire motion trajectory to satisfy the full-body dynamical model, as shown in Equation (4).

(3). In terms of kinematics, during the optimization process of the robot, the maximum extension of the robot’s end-effector should less than the length of the robot arm or leg and should meet the forward kinematics equation [21].

(7) $$\begin{array}{cc} \; & r\leq L_{max}\\ \; & r=g(q_{j}) \end{array}$$

(4). During the optimization process, we found that when the robot falls in a non-dynamic environment, the end points of the robot’s arms or legs are prone to mold piercing when colliding with the ground, as shown in Figure 3. Therefore, it is necessary to set the vertical distance $p_{i}^{z}>0$ of the robot’s collision point.

(5). When the robot makes contact with the ground, we want no slippage to occur. Based on Posa’s contact complementarity constraint equations [27], we can obtain the relationship between the contact position, contact force, and contact velocity, as described below, where ξ is a slack coefficient to encourage convergence.

(8) $$\begin{array}{cc} \; & p_{c}*f_{n}⩽\xi \end{array}$$

(9) $$\begin{array}{cc} \; & {\dot{p}}_{c}*f_{n}⩽\xi \end{array}$$

(6). In [28], the robot’s sole was simplified to four support points, with each support point corresponding to a tetrahedron that forms a relatively complex friction cone constraint. In this paper, a simplified model is adopted by approximating each contact location to a single point. Based on the friction relationship, the following equations can be obtained, where $F_{N}$ and $F_{f}$ are optimization variables from the inputs above.

(10) $$\mu_{f}F_{N}\leq F_{f}$$

## 3. Controller

### 3.1. Air Stage Controller

Through previous trajectory optimizations, we can optimize the state variables and control inputs of the robot during the falling process. These parameters are essential for setting the motion controller. During the air stage, due to differences between the simulation model and the actual physical model, there will be deviations in the position and orientation of the robot’s center of mass during motion. Therefore, designing an effective motion controller to minimize these deviations becomes crucial. The PD controller has the function of simple parameters and easy implementation, so we set the following control equations

(11) $$\begin{array}{cc} \; & {\ddot{p}}_{comout}=k_{p_{c}}\left(p_{a}-p_{d}\right)+k_{d_{c}}\left({\dot{p}}_{a}-{\dot{p}}_{d}\right)\\ \; & {\dot{w}}_{comout}=k_{R_{c}}log\left(R_{a}R_{d}\right)+k_{w_{c}}\left({\dot{\alpha }}_{a}-{\dot{\alpha }}_{d}\right) \end{array}$$

(12) $$\begin{array}{cc} \; & {\ddot{p}}_{endout}=k_{p_{e}}\left(p_{enda}-p_{endd}\right)+k_{d_{e}}\left({\dot{p}}_{enda}-{\dot{p}}_{endd}\right)\\ \; & {\dot{w}}_{comout}=k_{R_{e}}log\left(R_{enda}R_{endd}\right)+k_{w_{e}}\left({\dot{\alpha }}_{enda}-{\dot{\alpha }}_{endd}\right) \end{array}$$

where *p* represents the position, *R* represents the orientation, subscript *a* represents the actual value, subscript *d* represents the desired actual value, and end represents the endpoint of arms or legs. The variables $k_{p_{c}}$, $k_{d_{c}}$, $k_{R_{c}}$, $k_{w_{c}}$ represent the corresponding coefficients and have the same meaning as the coefficients in Equation (12).

### 3.2. Landing Controller

During a robot’s walk or run, the center of mass is usually placed at the hip joint center; however, the robot requires a more precise position during falls. Therefore, we use the actual link lengths and mass distributions to solve for the corresponding center-of-mass position.

(13) $$p_{a}=\frac{\sum_{i=1}^{N_{n}}m_{i}p_{i}\left(q\right)}{\sum_{i=1}^{N}\left(m_{i}\right)}$$

where $N_{n}$ is the total number of links, $m_{i}$ is the mass of the *i*th link, and $p_{i}\left(q\right)$ represents the position of the *i*th link in the world coordinate frame.

Although the aforementioned trajectory optimization method solves for the impact force of the robot’s contact points during landing, this is done without considering external disturbances and lacks some robustness. In other words, since the landing time is relatively short, designing a method that can efficiently and quickly respond to such impact forces becomes very important. Model predictive control needs high precision in modeling, but optimization is computationally intensive and time-consuming. Also, different cost functions need to be designed for various scenarios, limiting generalizability. Since a robot’s fall from the air and contact with the ground happens very quickly, a fast-responding algorithm is needed. Here, we refer to the landing control method proposed in [29]; according to the Newton–Euler laws of motion, we can obtain the following:

(14) $$\begin{array}{cc} \; & m{\dot{p}}_{com}=F_{all}-mg\\ \; & \dot{L}=n-c\times F_{all} \end{array}$$

where *m* denotes the mass of the robot, $p_{com}$ is the position of the center of mass, *c* is the position from the contact point to the center of mass, *L* is the angular momentum about the center of mass, and $F_{all}$ and *n* represent the force and moment, respectively, exerted on the robot by the environment, expressed in the world frame. Similar to [30], we approximate the angular momentum equation of the robot’s center of mass as follows:

(15) $$L=I_{all}\dot{q}\approx I_{base}w\approx Iw$$

where $I_{all}$ is the angular part of the centroidal momentum matrix; $\dot{q}$ is the angular velocity of all joints, including the floating base; $I_{base}$ is the matrix block corresponding to the base coordinate in $I_{all}$; *w* is the angular velocity of the base link; and *I* is a constant and diagonal approximation of $I_{base}$. Combining Equations (14) and (15) and neglecting the effect of the moment, we can obtain the following equation:

(16) $$\left[\begin{array}{c} I\\ c\times \end{array}\right]f=\left[\begin{array}{c} m({\ddot{p}}_{com}+g)\\ I\dot{w} \end{array}\right]$$

We set the force and torque when the robot lands as

(17) $$N_{d}=\left[\begin{array}{c} m({\ddot{p}}_{comout}+g)\\ I{\dot{w}}_{comout} \end{array}\right]$$

Let

(18) $$M=\left[\begin{array}{c} I\\ c\times \end{array}\right]$$

We can express Equation (16) as a form of a quadratic program (QP), where $\alpha_{1}$ and $\alpha_{2}$ represent the corresponding weight coefficients, the rightmost term in the equation means that the expected landing impact force of the robot is minimized.

(19) $$\begin{array}{cc} \; & \min_{f}\alpha_{1}{\left(Mf-N_{d}\right)}^{2}+\alpha_{2}f^{2}\\ \; & \;s.t.\mu *F_{N}⩽F_{f} \end{array}$$

### 3.3. Spring–Damper Controller

**Figure 4:**
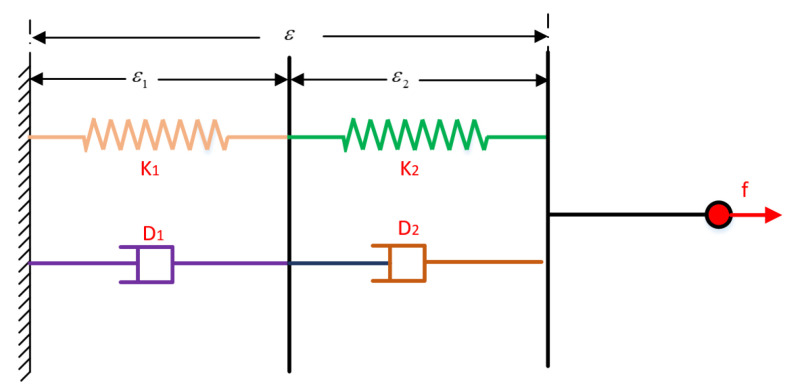
Spring–damper model.

Using the method above, we have optimized for the impact force during the robot’s landing. However, since our robot is position controlled, we need to convert this to corresponding joint angles. To do so, we adopt a dual spring–damper model as shown in Figure 4. Where $k_{1}$, $k_{2}$, $D_{1}$, and $D_{2}$ are the spring and damper coefficients, respectively.

Let ε be the overall deformation of the robot after being subjected to an external force *f*, where $\varepsilon_{1}$ and $\varepsilon_{2}$ are the deformations on the left and right sides, respectively. Then according to Hooke’s law, we can obtain:

(20) $$\begin{array}{cc} \; & \varepsilon =\varepsilon_{1}+\varepsilon_{2}\\ \; & f=k_{1}\varepsilon_{1}+D_{1}{\dot{\varepsilon }}_{1}=k_{2}\varepsilon_{2}+D_{2}{\dot{\varepsilon }}_{2} \end{array}$$

Applying a Laplace transformation to the second term of Equation (20) yields

(21) $$\begin{array}{cc} \; & \varepsilon_{1}=\frac{f}{k_{1}+D_{1}s}\\ \; & \varepsilon_{2}=\frac{f}{k_{2}+D_{2}s} \end{array}$$

Substituting Equation (21) into the first term of Equation (20) and taking the inverse Laplace transform, we obtain

(22) $$k_{1}k_{2}\varepsilon +\left(k_{2}D_{1}+k_{1}D_{2}\right)\dot{\varepsilon }+\ddot{\varepsilon }=f\left(k_{1}+k_{2}\right)+\dot{f}\left(D_{1}+D_{2}\right)$$

Let the state variable of the spring–damper model be $\chi ={\left[\begin{array}{c} f,\varepsilon ,\dot{\varepsilon } \end{array}\right]}^{T}$, where the control input $u=\ddot{\varepsilon }$; then the state equation of this spring–damper model can be written as:

(23) $$\dot{\chi }=A\chi +B\nu$$

(24) $$\frac{d}{dt}\left[\begin{array}{c} f\\ \varepsilon \\ \dot{\varepsilon } \end{array}\right]=\left[\begin{array}{ccc} \frac{k_{1}+k_{2}}{D_{1}+D_{2}} & \frac{k_{1}k_{2}}{D_{1}+D_{2}} & \frac{k_{1}D_{2}+k_{2}D_{1}}{D_{1}+D_{2}}\\ 0 & 0 & 1\\ 0 & 0 & 0 \end{array}\right]$$

Based on Equation (23), we can obtain the relationship between the desired state variable and the desired input as follows:

(25) $$\dot{\chi_{d}}=A\chi_{d}+B\nu_{d}$$

Subtracting Equation (23) from (25), we have

(26) $$\Delta \dot{\chi }=A\Delta \chi +B\Delta \nu$$

The state feedback controller is given by

(27) Δν=−KΔχ

where $k={\left[k_{1};k_{2};k_{3}\right]}^{T}$, which can be obtained through the LQR method. Define the cost function as

(28) $$\begin{array}{cc} J= & \frac{1}{2}\int_{0}^{\infty }\left(\Delta \chi^{T}Q\Delta \chi +\Delta \nu^{T}R\Delta \nu \right)dt\\ = & \frac{1}{2}\int_{0}^{\infty }\Delta \chi^{T}\left(Q+K^{T}RK\right)\Delta \chi dt \end{array}$$

where *Q* and *R* are the weight matrices of the state variables *X* and the input variable *u*, respectively. Let $K=R^{-1}B^{T}P$, where *P* can be obtained through the Riccati equation:

(29) $$A^{T}P+PA+Q-PBR^{-1}B^{T}P=0$$

Through Equations (28) and (29), we can get the values of the gain coefficient $k={\left[k_{1};k_{2};k_{3}\right]}^{T}$; then the end change can be expressed as follows:

(30) $$\ddot{\Delta \varepsilon }=-k_{1}\left(f_{real}-f\right)-k_{2}\Delta \varepsilon -k_{3}\dot{\Delta }\varepsilon$$

## 4. Simulation and Experiment

### 4.1. Simulation Platform

**Figure 5:**
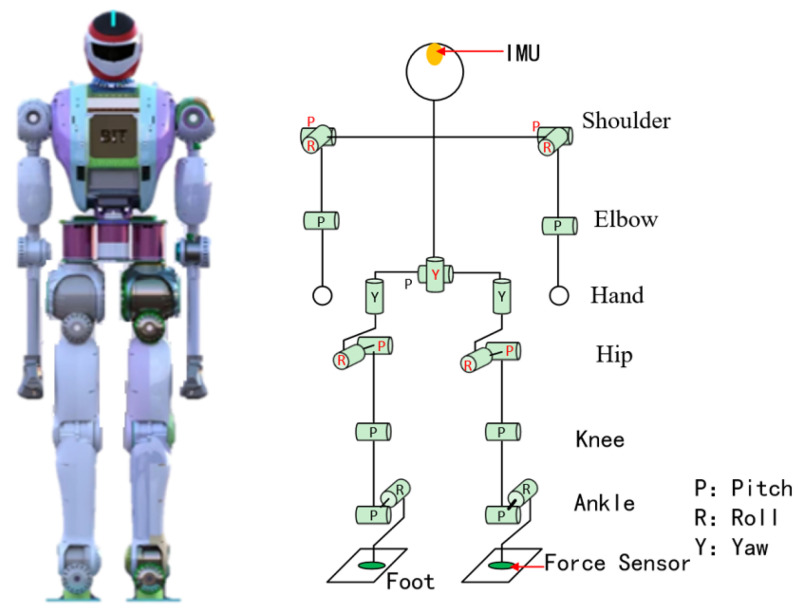
Snapshots of the humanoid robot. The left side represents a three-dimensional view of the robot, and the right side depicts a schematic diagram of the robot’s joints.

The simulation platform used in this paper is a bipedal robot independently developed by our laboratory, as shown Figure 5. The robot weighs 50 kg in total and has 20 degrees of freedom, including 6 degrees of freedom in the legs, 2 degrees of freedom in the waist, and 3 degrees of freedom in the arms. The specific dimensions and parameters are shown in Table 1.

### 4.2. Trajectory Optimization

**Figure 6:**
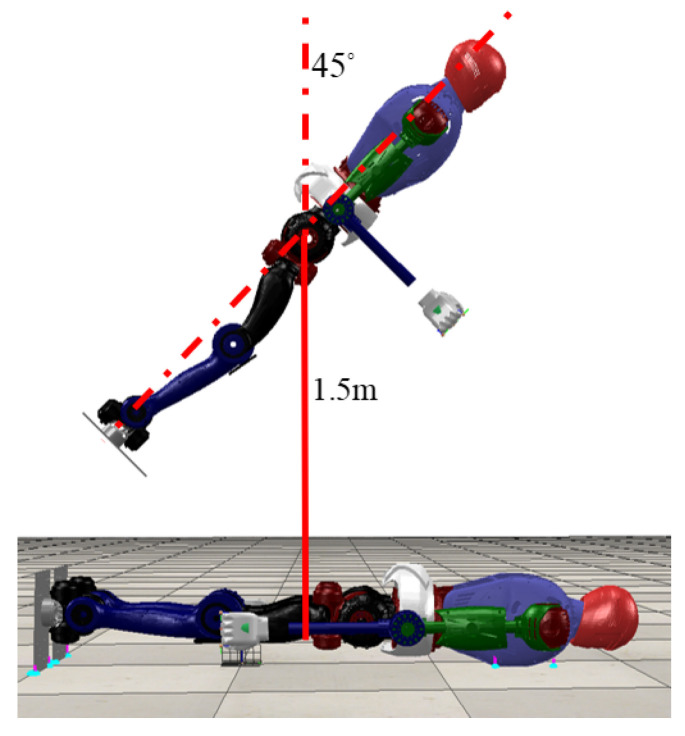
The robot free-falls through the air and in the final state lies flat on the ground.

The robot’s falling trajectory was optimized in MATLAB, while the robot’s kinematics and dynamics were generated by the open-source software FROST [31]. The optimization library used consulted Matthew Kelly [32,33]. In the optimization process, we first follow the first constraint introduced in Section 2.3 of the article to give the upper and lower bounds of the robot’s state variables, motion time, state input, and so on. To shorten the optimization time and avoid local optima in the first optimization process, we give a free-fall trajectory as an initial trajectory, which is a simple trajectory that does not satisfy the dynamic constraints but can constrain the optimization result to an ideal reliable value range, as shown in Figure 6. Additionally, we selected the trapezoidal method as the interpolation approach. Obviously, the initial reference trajectory from the first optimization did not satisfy the dynamics equations. So after this, we put the optimized reference trajectory into the estimate, performed a second optimization, and repeated this process until the optimal trajectory appeared.

### 4.3. Simulation

We perform the simulations using Coppeliasim and MATLAB. In Coppeliasim, the robot’s physical engine is Bullet 2.78 and the control period is 5 ms. The initial pitch angle of the robot is $45^{\circ }$, the distance from the ground is 1.5 m, the ground friction coefficient is 0.75, and the other parameters are introduced in Table 2, which appears at the end of the article. We set two different sets of state variables to compare the optimization results: the first set of optimization variables are as shown in Equation (1), and the second set of optimization variables are as shown in Equation (2). The other constraints are kept consistent. In addition, we also simulated the effect of adding the controller to the robot when it landed.

#### 4.3.1. Simple State Variables

**Figure 7:**
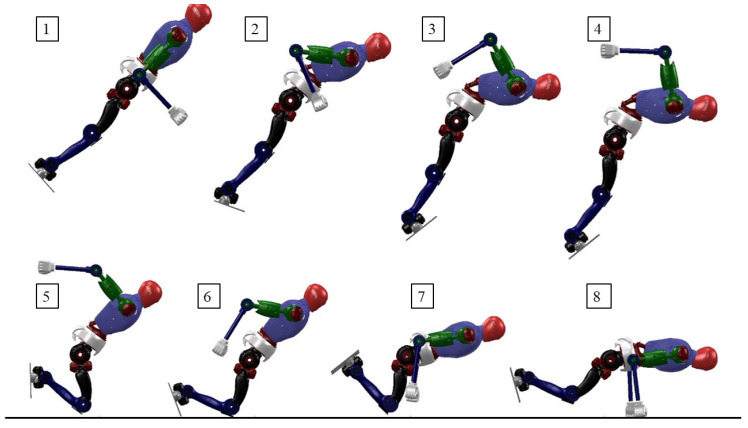
Snapshots of the robot falling from the air without contact point information in the state variables.

As described above, we used the equation in (1) for the optimization variables. Figure 7 shows the optimized robot motion states obtained using this method. As can be seen from Figure 7(1)–(4), in order to reduce the landing speed, the robot swings its arms backward. Figure 7(5)–(8) show that after the robot lands, its knees quickly touch the ground and its arms also start to find the landing position. Figure 7(8) is the final state, and it can be seen that without the constraint of touchdown information, the robot has problems such as flipping over backward and unbalanced ground contact, and the overall optimized motion exhibits unreasonable phenomena.

#### 4.3.2. Extended State Variables

**Figure 8:**
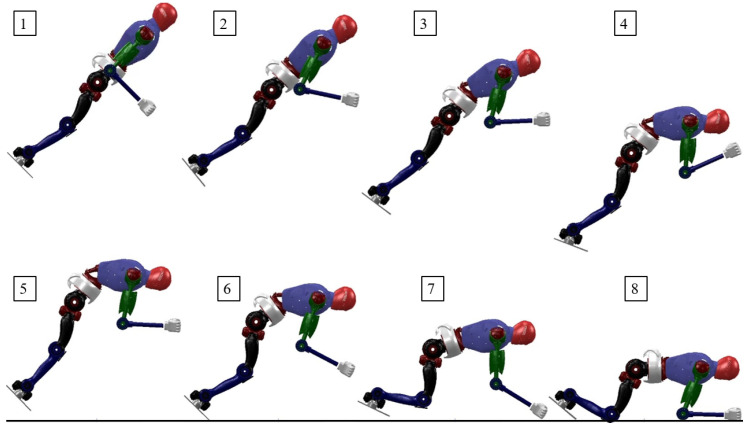
Snapshots of the robot landing without damping controller.

In the second set of trajectory optimization experiments, we optimized using extended state variables. The optimized robot motion states obtained are shown in Figure 8. Compared to Figure 7, the state variables contain touchdown information. It can be seen that when in the air, the robot stretches its arms and legs straight while bending its waist, which is conducive to adapting when contacting the ground; after the front end of the foot touches the ground, the robot immediately bends its knees to reduce the ground impact while the arms touch the ground to share the pressure. After the robot’s hands or knees make contact with the ground, the whole body keeps balance.

#### 4.3.3. Extended State Variables and Control

**Figure 9:**
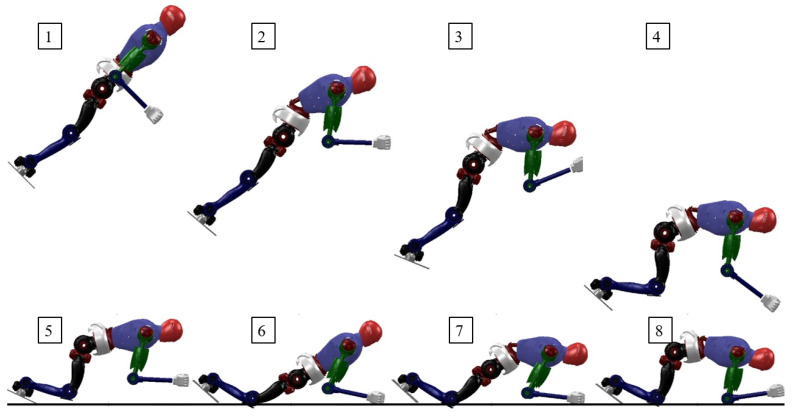
Snapshots of the robot landing with the damping controller.

Although the trajectories optimized using extended state variables conform to the full-body dynamics model, the impact force during the robot’s landing remains large. To solve this problem, we incorporated the motion controller described above. When the robot is moving in the air, simple PD control is used. However, when the robot lands, the landing controller is engaged. Figure 9(5)–(8) shows the effect of it. Once contact with the ground is detected, the robot swings its arms backward, presses down its body, and moves forward. Near the end of landing buffering, in order to maintain an overall balanced posture, the robot’s upper body moves upward and recovers to a four-point landing state.

#### 4.3.4. Graphical Analysis

**Figure 10:**
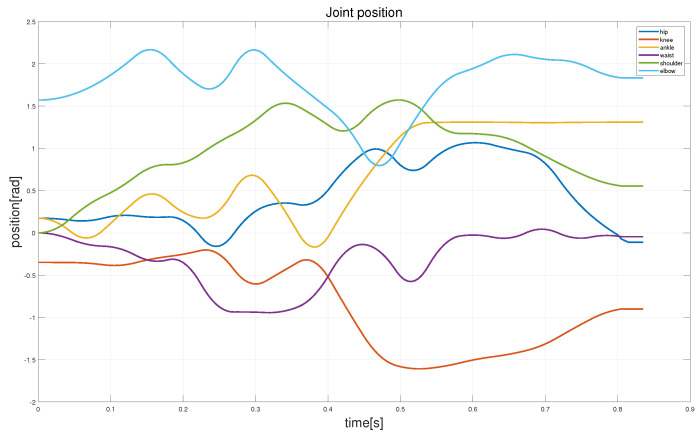
The joint trajectory curve optimized by our method. Owing to the bilateral symmetry between the legs and arms, only the joint angle profiles of the right leg, waist, and arm of the robot are displayed.

Due to the significant changes in the z- and y-directions during the robot’s fall, this study primarily considers the hip, knee, ankle, waist, shoulder, and elbow joints. Figure 10 shows the joint angle trajectories of the robot joints optimized using the aforementioned trajectory optimization method. It can be observed from the figure that the joint angle trajectories vary smoothly without any anomalous values.

**Figure 11:**
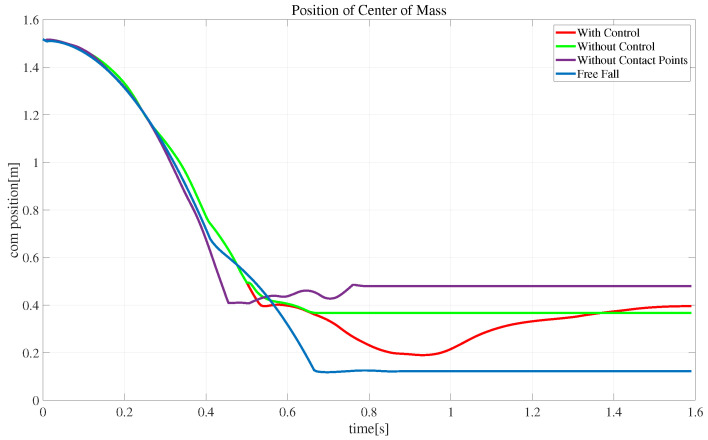
Snapshots of the centroid position of the robot falling from the air.

**Figure 12:**
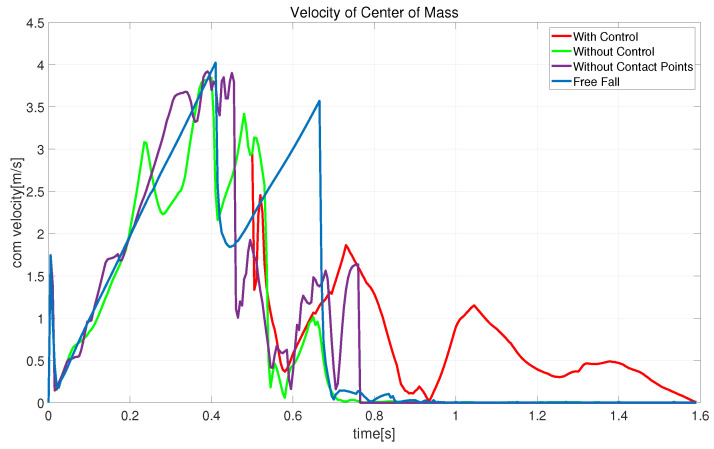
Snapshots of the centroid velocity of the robot falling from the air.

**Figure 13:**
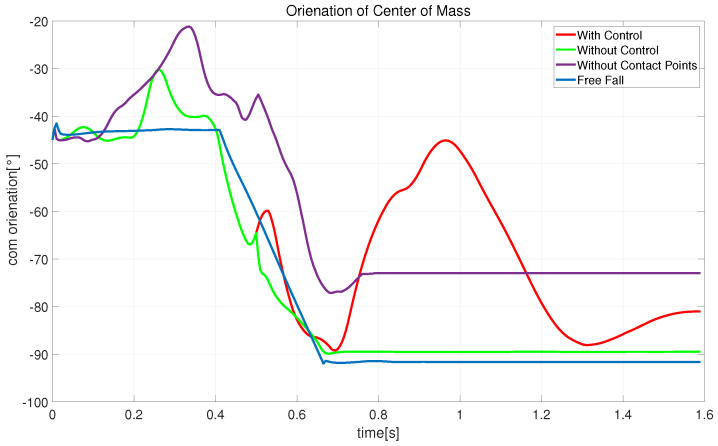
Snapshots of the centroid orientation of the robot falling from the air.

Figure 11, Figure 12 and Figure 13 show schematic diagrams of the robot’s actual center-of-mass position, velocity, and orientation, respectively, when falling from the air. The trajectory plots include the cases of extended state variables and control (with control), extended state variables (without control), simple state variables (without contact points), and free-fall motion. The other snapshots are similar. The red curve in Figure 11 shows that after adding the landing controller, the robot’s center-of-mass trajectory continues moving downward and then recovers to a stable state, consistent with the motion in Figure 9. In Figure 12, the free-fall motion velocity curve is blue and has a maximum velocity reaching 4.02 m/s. The purple curve indicates optimization with no contact point information in the state variables: the robot lands after 0.76 s with a maximum velocity of 3.92 m/s. Comparing the red and fluorescent curves (without control), the speed of the robot drops directly from 3.1315 m/s to 0.18185 m/s within 0.04 s after landing, while with control, the velocity decreases from 2.9311 m/s to 1.3363 m/s. This demonstrates that the proposed landing controller provides good shock absorption. Figure 13 shows the orientation of the robot during landing, and it can be seen that after touchdown, the landing controller starts to take effect, demonstrating the effectiveness of the proposed method.

**Figure 14:**
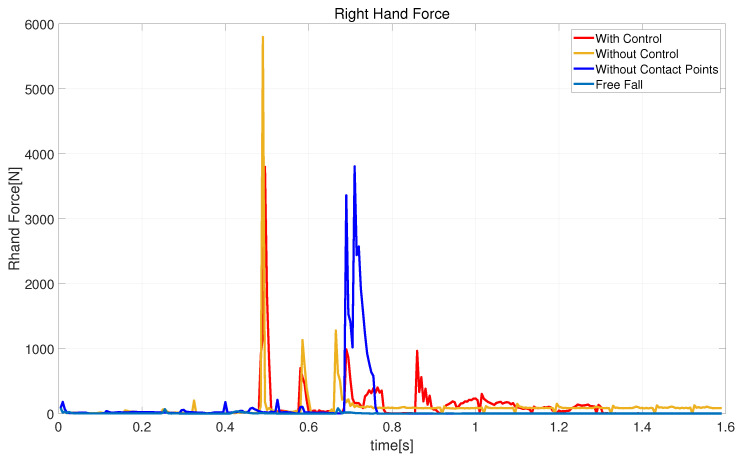
Schematic diagram of the impact force generated by the robot’s right hand hitting the ground.

Figure 14 shows a schematic diagram of the impact force on the robot’s right hand when striking the ground. Since in free-fall motion we do not want the robot’s arms to contact the ground, the impact force is almost 0 during landing. Compared to no landing controller and trajectory optimization without contact point information, the red curve represents the use of control during the fall and has a maximum impact force of 5797 N at the instant of touchdown. Approximately 0.1 s later, a secondary impact occurs, but this time the impact force is 709.2 N, which is less than the 1132 N experienced without a landing controller.

**Figure 15:**
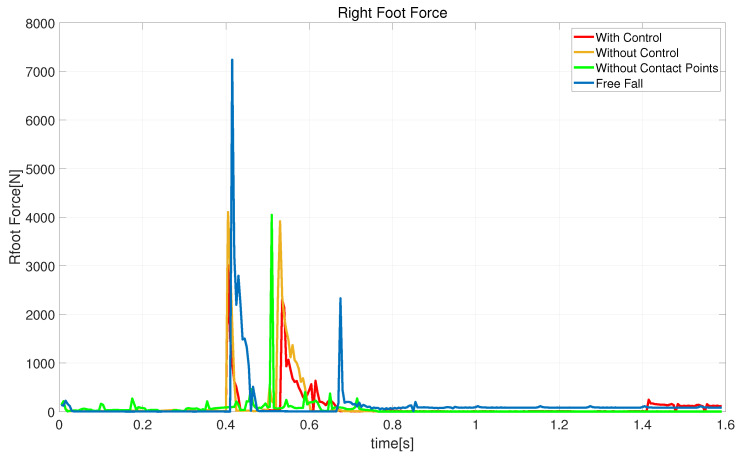
Schematic diagram of the impact force generated by the robot’s right foot hitting the ground.

Figure 15 shows the ground reaction force on the robot’s right foot: it can be seen that free-fall motion has the most significant impact force. After adding the landing controller, the robot’s first impact force is reduced to about 4100 N, and the second impact force is reduced to 2300 N. Comparing without control and without contact points, it can be seen that the impact effects of both are similar. These data demonstrate that the proposed methods can handle ground impact forces effectively.

### 4.4. Experiment

**Figure 16:**
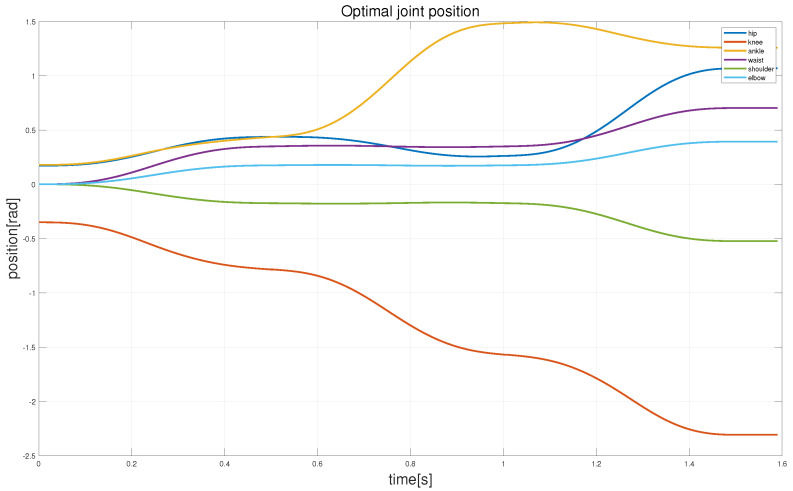
Robot joint position.

**Figure 17:**
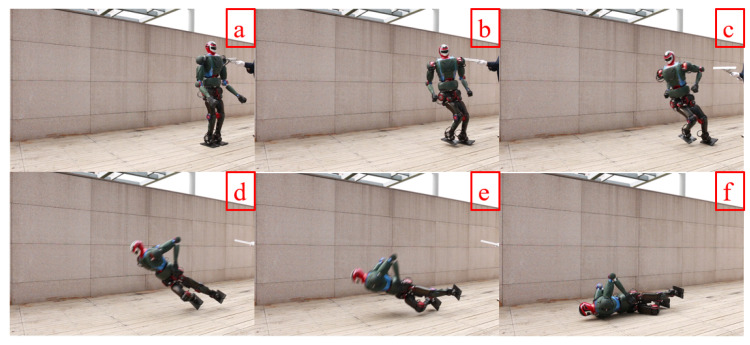
The robot falls on its side, and in Figures (**a**,**b**) an external force is applied to the robot, Figures (**c**,**d**) represents that the robot completes this protection action according to the optimized trajectory and PD control, Figures (**e**,**f**) indicates that the landing controller takes effect after the robot reaches the ground.

Falling from the air is a very dangerous maneuver for the robot and requires various safety equipment. However, we were unable to complete this experiment at this time since the hardware environment is still being set up. To validate the method proposed in this paper, we conducted an experiment outdoors using the example of the robot falling forwards to the right. We completed this experiment using trajectory optimization and motion control methods. Figure 16 shows the joint angle profiles of the robot optimized using the aforementioned optimization approach. During the motion, we applied a lateral force of approximately 150 N to the robot for 0.3 s. When the lateral push force exceeded the robot’s self-adjustment range, it had to fall. Our robot detected the falling direction and threshold based on the method proposed in [34]. Upon detecting the fall, the robot’s right leg quickly lifted up and the waist joint immediately twisted to brace for impact with the ground. Since the robot’s hands are quite delicate, to avoid damage during the collision, we swung the arms upwards during the fall. This minimized the impact on the hands; the final effect is shown in Figure 17.

## 5. Conclusions

This paper optimizes a protection trajectory for a robot falling from the air. Compared with traditional trajectory optimization methods, this paper abandons the strategy of humanoid robots of working from the high-level motion task strategy space to the center-of-mass space and then to the whole-body joint space. Rather, we establish a relationship between the high-level motion task strategy space and the whole-body joint space. Moreover, this paper adds the robot’s contact point information to the state variables, enabling it to utilize reference contact information to avoid phenomena that do not comply with contact dynamics during trajectory optimization. A PD controller is added during the robot’s flight phase to control the position and direction of the center of mass or the endpoints. During the robot’s contact phase, according to the center-of-mass dynamics model, the contact force is optimized. Assuming that force sensors are installed at the endpoint parts of the robot, then according to the actual applied force and the optimized applied force, we can use a damping controller to calculate the movement of the endpoint to finally put the above results into an inverse kinematics optimization based on QP to obtain the joint angle changes required to desired forces. The simulation and hardware experiment results show that by combining trajectory optimization and motion control methods, the robot can safely fall to the ground. Compared to simple free-fall motion or only simple state variables, this method can effectively buffer the impact force.

## 6. Future Work

In the future, we will implement online motion trajectory optimization of the robot and deploy it to the physical prototype to further prove the effectiveness of this method.

## Figures and Tables

**Table 1:** The parameters of our robot.

Parameter	Size	Mass
Thigh	361 (mm)	7.36 (kg)
Shank	330 (mm)	5.12 (kg)
Boom	350 (mm)	4.15 (kg)
Jib	360 (mm)	2.3 (kg)
Others	─	31.07 (kg)
Total Mass	─	50 (kg)

**Table 2:** The control parameters of the simulation.

Parameter	Value	Parameter	Value	Parameter	Value
$w_{1}$	0.1	$k_{p_{e}}$	diag([10 850 1760])	$k_{2}$	200
$w_{2}$	0.8	$k_{d_{e}}$	diag([0.005 27 50])	$D_{1}$	1500
$w_{3}$	0.005	$k_{R_{e}}$	diag([5 150 1000])	$D_{2}$	1800
ξ	0.03	$k_{w_{e}}$	diag([0.005 52.3 65])	Q	diag([1 1 1])
$k_{p_{c}}$	diag([10 1000 2000])	$\alpha_{1}$	0.52	R	0.0001
$k_{d_{c}}$	diag([0.01 12.7 45])	$\alpha_{2}$	1	m	50
$k_{R_{c}}$	diag([2 100 750])	μ	0.75		
$k_{w_{c}}$	diag([0.002 10.0 25])	$k_{1}$	210,000		

## Data Availability

Not applicable.
